# Free-breathing myocardial T2* mapping using GRE-EPI and MOCO for myocardial and hepatic iron overload assessment: a multi-centre study

**DOI:** 10.1186/1532-429X-18-S1-Q31

**Published:** 2016-01-27

**Authors:** Ning Jin, Juliano L Fernandes, David Firmin, Clerio F Azevedo, Juliana Serafim da Silveira, George Lathra Mathew, Nathan Lamba, Sharath Subramanian, Dudley J Pennell, Subha V Raman, Orlando P Simonetti

**Affiliations:** 1 MR R&D, Siemens Healthcare, Columbus, OH USA; 2 Radiologia Clinica de Campinas, Jose Michel Kalaf Research Institute, Campinas, Brazil; 3 NIHR Cardiovascular Biomedical Research Unit, Royal Brompton and Harefield NHS Trust, London, United Kingdom; 4 Clinica de Diagnositco Por Imagem (CDPI), Rio de Janeiro, Brazil; 5 Davis Heart & Lung Research Institute, The Ohio State University, Columbus, OH USA; 6 Department of Internal Medicine - Division of Cardiovascular Medicine, The Ohio State University, Columbus, OH USA

## Background

T2* measurement is widely used in the assessment of patients at risk for cardiac and hepatic iron overload (1,2). The conventional breath-hold, ECG-triggered, segmented, multi-echo gradient echo sequence (BH MGRE) used for myocardial T2* quantification (3) is very sensitive to respiratory motion and may not be feasible in patients who are unable to breath-hold. To overcome this limitation, we developed a free-breathing myocardial T2* mapping approach (4). The goal of this study was to investigate the effectiveness of the new technique in patients referred for iron overload assessment at 4 different centers across the world.

## Methods

The free-breathing T2* mapping technique uses a gradient-echo, echo-planar imaging sequence that acquires single-shot images at different TE's in different heartbeats. Automatic non-rigid motion correction is used to register images at different TEs prior to T2* estimation (FB MOCO GRE-EPI).

A total of 95 patients (52 males, age 34.2 ± 19.7 years, range 7 to 81), referred for CMR evaluation of hepatic and myocardial iron (38 from Campinas, Brazil, 28 from London, UK, 20 from Rio de Janeiro, Brazil, 9 from Columbus, OH, US) were studied. A single mid-ventricular short axis slice was acquired in all patients. The quality of T2*-weighted images was scored independently by 2 experienced observers blinded to the image acquisition strategy. A 5-point scoring system was used ranging from 1 very poor to 5 excellent image quality. In those cases in which both techniques were judged by both reviewers to have at least adequate image quality, the mean T2* measured in the interventricular septum and liver were compared between the two techniques.

## Results

**Figure 1:**
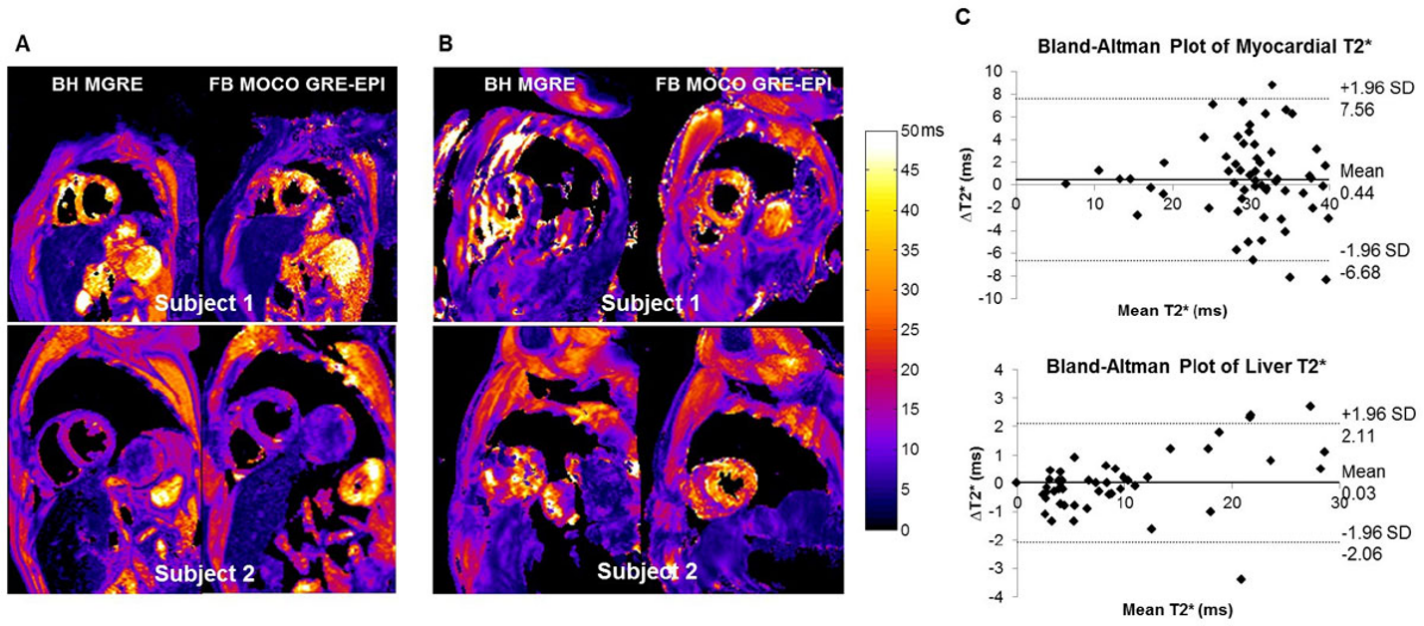
(A) Examples of good T2* maps from BH MGRE and FB MOCO GRE-EPI in two subjects able to breath hold. Subject 1 has normal myocardial T2* and mild hepatic iron overload. Subject 2 has mild myocardial and moderate hepatic iron overload. (**B**) In two subjects unable to breath hold, FB MOCO GRE-EPI improves T2* map quality. Subject 1 has normal myocardial and hepatic T2*. Subject 2 has normal myocardial T2* and mild hepatic iron overload. (**C**) Comparisons between the mean T2* measured in the intraventricular septum and the liver from the two techniques demonstrated good agreement.

Myocardial iron overload (T2*<20 ms) was present in 14 patients, and hepatic iron overload (T2*<11.4 ms) in 64 patients based on BH MGRE results. All patients who had myocardial iron overload also had hepatic iron overload. The average image quality scores are shown in Table. There were no significant differences between the quality scores of the BH and FB techniques by either reviewer. 24 BH MGRE studies (35.7% of all the patients) were scored below average or very poor due to failed breath-hold, while only 14 FB MOCO GRE-EPI studies (14.7% of all the patients) were less than adequate (Fig 1A-B). In the 60 patients with at least adequate image quality by both techniques, the intra-class correlation coefficient for myocardial and hepatic T2* were 0.879 and 0.990 respectively (p < 0.001), indicating very consistent T2* measurements. Bland-Altman plots (Fig 1C) demonstrate good agreement.

## Conclusions

This study shows that the free-breathing T2* mapping using MOCO GRE-EPI enables consistent image quality and accurate myocardial and hepatic T2* measurements in patients referred for iron overload assessment at different centers in different parts of the world.

**Table 1:** Image Quality Score

	Reviewer 1	Reviewer 2
BH MGRE	3.56 ± 1.33	3.74 ± 1.27
FB MOCO GRE-EPI	3.47 ± 0.85	3.58 ± 0.97
